# NORDIC denoising on VASO data

**DOI:** 10.3389/fnins.2024.1499762

**Published:** 2025-01-06

**Authors:** Lasse Knudsen, Luca Vizioli, Federico De Martino, Lonike K. Faes, Daniel A. Handwerker, Steen Moeller, Peter A. Bandettini, Laurentius Huber

**Affiliations:** ^1^Center of Functionally Integrative Neuroscience (CFIN), Aarhus University, Aarhus, Denmark; ^2^Sino-Danish Center for Education and Research (SDC), University of Chinese Academy of Sciences, Beijing, China; ^3^CMRR, University of Minnesota, Minneapolis, MN, United States; ^4^FPN, Maastricht University, Maastricht, Netherlands; ^5^Section on Functional Imaging Methods, NIH, National Institute of Mental Health, Bethesda, MD, United States; ^6^Functional Magnetic Resonance Imaging (FMRI) Core, NIH, National Institute of Mental Health, Bethesda, MD, United States

**Keywords:** NORDIC, denoising, VASO, laminar fMRI, submillimeter resolution

## Abstract

The use of submillimeter resolution functional magnetic resonance imaging (fMRI) is increasing in popularity due to the prospect of studying human brain activation non-invasively at the scale of cortical layers and columns. This method, known as laminar fMRI, is inherently signal-to-noise ratio (SNR)-limited, especially at lower field strengths, with the dominant noise source being of thermal origin. Furthermore, laminar fMRI is challenged with signal displacements due to draining vein effects in conventional gradient-echo blood oxygen level-dependent (BOLD) imaging contrasts. fMRI contrasts such as cerebral blood volume (CBV)-sensitive vascular space occupancy (VASO) sequences have the potential to mitigate draining vein effects. However, VASO comes along with another reduction in detection sensitivity. NOise Reduction with DIstribution Corrected (NORDIC) PCA (principal component analysis) is a denoising technique specifically aimed at suppressing thermal noise, which has proven useful for increasing the SNR of high-resolution functional data. While NORDIC has been examined for BOLD acquisitions, its application to VASO data has been limited, which was the focus of the present study. We present a preliminary analysis to evaluate NORDIC’s capability to suppress thermal noise while preserving the VASO signal across a wide parameter space at 3T. For the data presented here, with a proper set of parameters, NORDIC reduced thermal noise with minimal bias on the underlying signal and preserved spatial resolution. Denoising performance was found to vary with different implementation strategies and parameter choices, for which we provide recommendations. We conclude that when applied properly, NORDIC has the potential to overcome the sensitivity limitations of laminar-specific VASO fMRI. Since very few groups currently have 3T VASO data, by sharing our analysis and code, we can compile and compare the effects of NORDIC across a broader range of acquisition parameters and study designs. Such a communal effort will help develop robust recommendations that will increase the utility of laminar fMRI at lower field strengths.

## 1 Introduction

**Figure 1:**
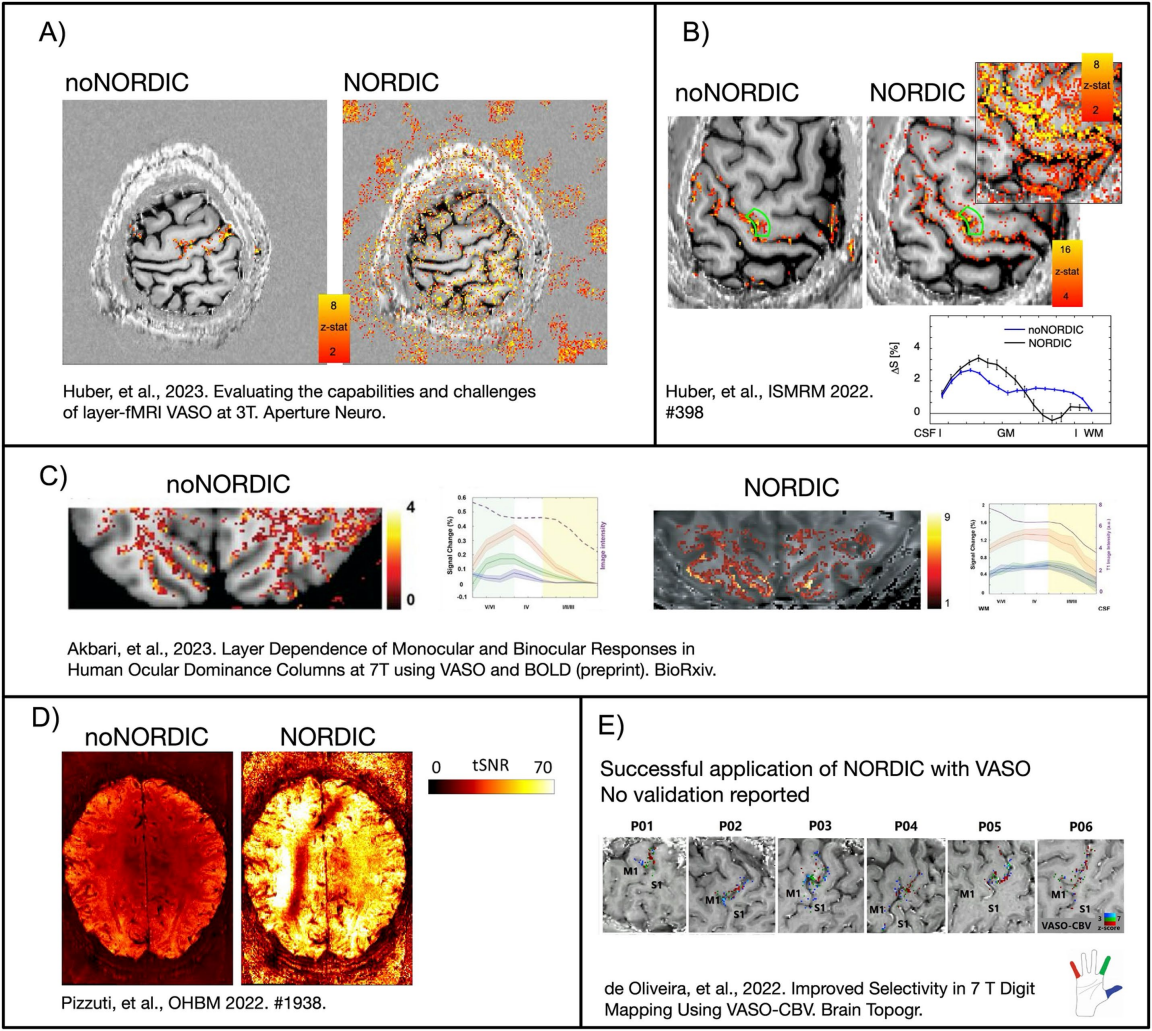
Example images of previous attempts to perform NORDIC with VASO. **(A)** NORDIC removed the activity of interest and introduced false-positive activity in patches outside the brain. It is suspected that this was due to the wrong application of noise estimates with overestimated variance of multiple contrasts as one time series. In this study, we want to explore the effect of noise floor estimates on NORDIC-VASO. **(B–D)** NORDIC boosts the sensitivity and tSNR by an order of magnitude. However, the layer profiles of beta scores are altered. It is suspected that the noise threshold estimation might have been overestimated by the different signal compositions of VASO **(B)** or by applying NORDIC with inappropriate coil-combined phase data **(D)**. This study explored the advantages and disadvantages of applying NORDIC-VASO in the complex-valued or magnitude-only domain. **(E)** The first NORDIC-VASO data published as a peer-reviewed journal article (denoising was performed on complex-valued data, separately on nulled and not-nulled time series, and without an appended noise volume). However, the aim of this study was not focused on the validation of NORDIC, and it did not report on the signal responses with and without denoising. In this study, we aimed to provide such validation and to provide recommendations to the field on how to apply NORDIC on VASO data with a minimum risk of adverse effects. Figures adapted with permission from Akbari et al. (2023), de Oliveira et al. (2023), Huber et al. (2023, 2022), and Pizzuti et al. (2022).

NORDIC is a PCA-based denoising method that may help boost the limited detection sensitivity of laminar fMRI through thermal noise suppression (Moeller et al., 2021; Vizioli et al., 2021). The merits and limitations of NORDIC for denoising fMRI data have previously been evaluated for BOLD acquisitions (Dowdle et al., 2023; Faes et al., 2024; Fernandes et al., 2023; Knudsen et al., 2023; Pfaffenrot and Norris, 2023 (ISMRM abstract); Vizioli et al., 2021), whereas accepted validations on VASO data are, to the authors’ knowledge, not available yet (but see Figure 1; Akbari et al., 2023; de Oliveira et al., 2023; Huber et al., 2023, 2022; Pizzuti et al., 2022). Compared with BOLD, VASO comes with an additional contrast (blood-nulled images), different phase data, and lower SNR. Furthermore, the inversion recovery nature of the VASO signal can result in CSF-dependent 180° phase skips across time appearing as high spatial resolution, temporal phase changes, which is a behavior that the original NORDIC implementation has not been optimized for. It is thus unclear how NORDIC should be executed on VASO data. Here, we tested the implementation of NORDIC denoising available in the study by Vizioli et al. (2021) across a wide parameter space to get a more intuitive feel for how to apply NORDIC on VASO data and to identify when it fails. We characterize the quality of NORDIC denoising by metrics of (1) temporal SNR (tSNR) and voxel-wise significance scores (*t*-values), (2) spatiotemporal structure of removed noise, (3) effect on response amplitudes compared to averaging non-denoised (noNORDIC) data, and (4) shape of laminar profiles.

## 2 Methods

### 2.1 Datasets

**Figure 2:**
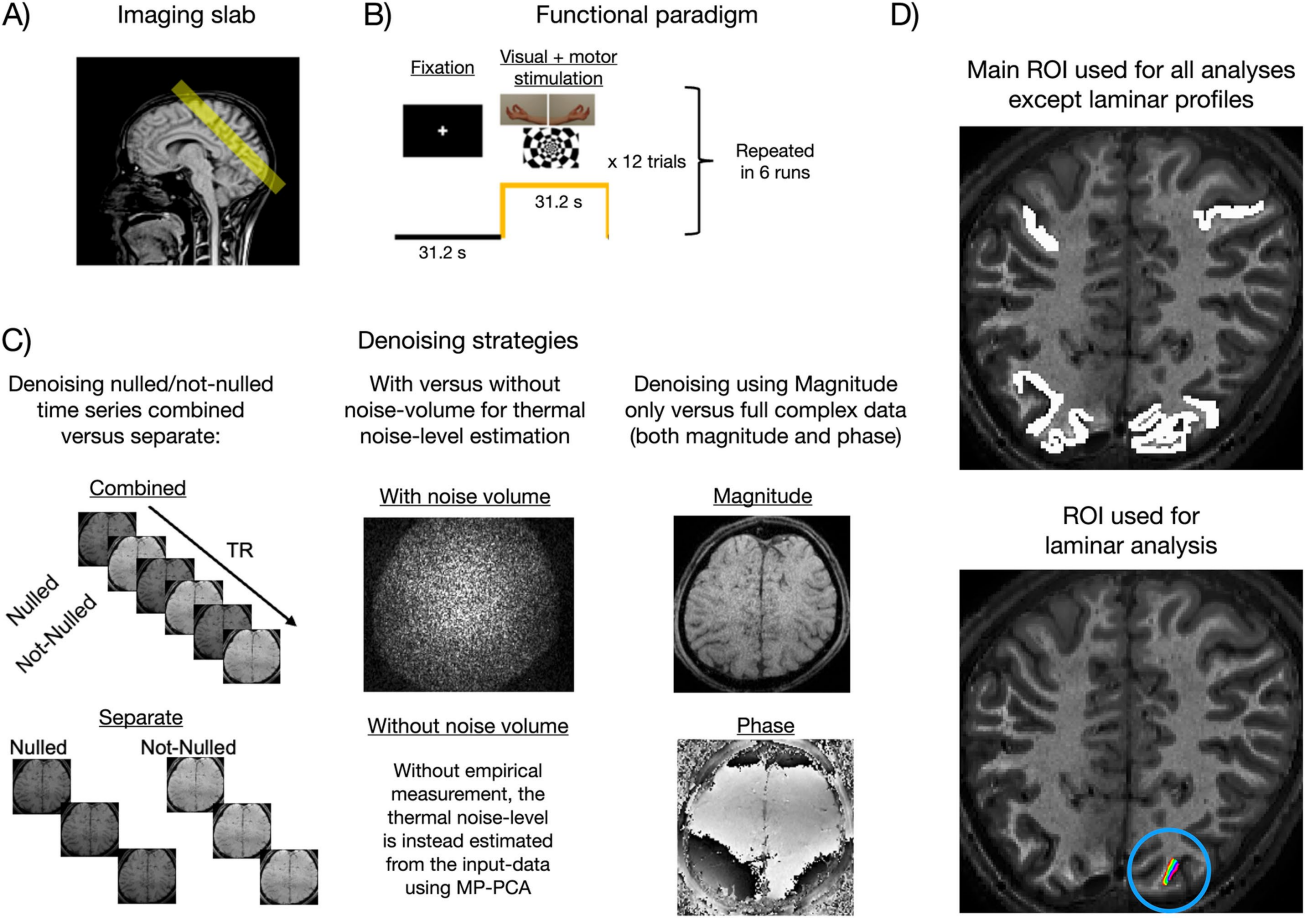
Methodological overview of the main experiment (3T). **(A)** The functional VASO imaging slab covered visual and sensorimotor cortices. **(B)** The paradigm consisted of resting blocks (fixation) alternating with concurrent visual and sensorimotor stimulation (Huber et al., 2023). **(C)** Illustration of the different NORDIC implementation strategies evaluated in the present study (see main text, Section 2.2). **(D)** The main ROI (upper panel) spanned multiple slices and contained more than 7,000 voxels in visual and sensorimotor areas. Responsive regions were roughly identified using BOLD activation maps, and the ROI was then coarsely drawn from there. The small-layered ROI (lower panel, within the blue circle), used for laminar analysis, spanned a single slice.

NORDIC was evaluated on a 3T segmented multi-echo VASO dataset (Huber et al., 2023, here we only used the first echo, TE = 12 ms), covering visual and motor cortices at 0.9 mm isotropic resolution (Figure 2A). The paradigm consisted of six 12-min runs of block-designed simultaneous visual stimulation and bilateral finger tapping (Figure 2B). We tested a 3T dataset rather than a 7T dataset because they are more dominated by thermal noise with a layer-resolution voxel size, making 3T VASO scans more challenging. If NORDIC improves VASO CNR at 3T, then advanced layer-fMRI methods become increasingly applicable, including at sites using 3T MRI systems. Furthermore, it presents a challenging test case given the CNR dependence on the success of low-rank denoising methods (Kay, 2022). Furthermore, it allowed for reasonable ground truth estimates at both the single-voxel level (after averaging across 72 trials, that supposedly mitigates most thermal noise) and the single-trial level (after averaging across an extensive activated area facilitated by combined visuomotor stimulation). Further parameters of this sequence were volume acquisition time for four sets of images (nulled and not-nulled combined, with two echoes each) = 5.2 s, inversion times (TI1/TI2) = 1072/1896 ms, phase partial Fourier = 6/8, matrix size = 216 × 216 × 12 (Huber et al., 2023), and skipped-CAIPI $6\cdot 1\times 3z_{2}$ (with 6 being the segmentation factor, 1 being the acceleration factor in the first phase encoding direction, 3 being the acceleration factor in the second phase encoding direction, and 2 being the CAIPI FOV shift in z). This notation is equivalent to segmentation factor 2, with in-plane GRAPPA 3, without GRAPPA *z*, and a prephased CAIPI in *z* of 2 (Stirnberg and Stöcker, 2021).

An additional 7T VASO dataset from a different subject was included to explore the effect of *g*-factor estimation strategies (described in Section 3.7). This dataset was acquired under the NIH-IRB (93-M0170, ClinicalTrials.gov: NCT00001360). Parameters were voxel size = 0.82 mm isotropic, TI1/TI2 = 1232/2386 ms, volume acquisition time (nulled and not-nulled combined) = 3.3 s, TE = 25 ms, $k_{y}$ -GRAPPA = 1, $k_{z}$ -GRAPPA = 3, phase partial Fourier = 6/8, and matrix size = 184 × 184 × 18.

### 2.2 Evaluating NORDIC implementation strategies

The VASO contrast is cerebral blood volume (CBV)-weighted and is believed to have high spatial specificity toward microvasculature. CBV weighting is obtained by applying an inversion pulse followed by image readout around the blood-nulling time, and the fMRI signal will thereby decrease in proportion to increases in CBV. These nulled images are interleaved with regular BOLD images (not-nulled images), which can be used to correct for positive BOLD contrast counteracting the negative CBV contrast in the nulled images (see details in Huber et al., 2014). As detailed below, it is somewhat unclear how NORDIC should be applied to these alternating contrasts. Furthermore, the VASO contrast has inherent high spatial frequency and temporally dependent phase fluctuations, which is not necessarily compatible with the complex-valued processing in NORDIC (for more background on the basic working principle of NORDIC, see layerfmri.com/nordic; Moeller et al., 2021; Vizioli et al., 2021). Therefore, we first evaluated a comprehensive spectrum of different NORDIC implementation strategies. Questions that we wanted to address were:

1. Should nulled and not-nulled volumes be denoised combined or separately (Figure 2C left column)? In the “combined” versions, NORDIC was applied to the full-time series where odd volumes are nulled and even volumes are not-nulled. For “separate” versions, the time series were split into nulled or not-nulled before denoising was applied to either of these individually. On the one hand, the combined version has the advantage of having twice the number of volumes, which should improve the ability of PCA to separate signal and noise components. On the other hand, the number of components required to describe the signal may be more than double in the combined case due to interactions between VASO and BOLD, thereby making it harder for PCA to dissociate signal and noise. Furthermore, although nulled and not-nulled images should have the same thermal noise levels, the *g*-factor and noise-level estimation could be hampered due to differences in CNR. In SS-SI VASO images, some tissue components with longer T1 values are expected to have alternating Mz phase polarities, while other tissue components with shorter T1 values are believed to have a constant phase. The locally specific jumps of 180° phase skips across individual voxels compared to others might make the implementation of NORDIC used here better suited for separate applications of given contrasts.
2. With or without an appended noise volume (Figure 2C middle column)? NORDIC allows the user to input a noise-only volume (typically acquired by the end of the run using the same sequence, but with the amplitude of the RF pulse set to 0). After *g*-factor normalization, the thermal noise level can be measured from this volume as the variance across space. If no noise volume is appended, the noise level is estimated from the data using MP-PCA (Veraart et al., 2016a, 2016b). The former option is theoretically preferred as it represents a more direct way of estimating the threshold. While this has been previously tested in BOLD data (Guidi et al., 2023, (ISMRM abstract)), here we empirically replicate this for VASO.
3. With complex-valued versus magnitude-only data (Figure 2C right column)? NORDIC can be applied using both magnitude and phase images (referred to here as complex versions) or magnitude images only. The complex-valued versions should theoretically have an advantage, partly because the data redundancy that low-rank denoising relies upon is increased when utilizing both the magnitude and phase information, and partly because the real and imaginary parts fulfill the assumption of Gaussian-distributed noise, whereas magnitude images have a Rician noise distribution (Gudbjartsson and Patz, 1995). The Rician distribution approaches a Gaussian shape when tSNR becomes sufficiently high. Since VASO data have relatively lower tSNR, we tested whether the inclusion of phase images makes a non-negligible difference in practice or if just using magnitude images is sufficient. In VASO, phase data are expected to be additionally challenged due to potential 180° phase skips of the inversion–recovery acquisition scheme. These phase skips are believed to vary locally (for individual CSF voxels within each NORDIC kernel) and across time based on the temporally varying adaptability of the inversion pulse in the presence of physiologically varying B0 in homogeneities.

We evaluated all $2^{3}=8$ implementation combinations of NORDIC and compared them with the non-denoised version (noNORDIC). For all analyses, we used the publicly available MATLAB (Mathworks Inc.) implementation of NORDIC,^1^ which comes in two flavors; “NORDIC.m” requires a noise-only volume and a *g*-factor map as inputs. Here, we focused on the other flavor, “NIFTI_NORDIC.m,” for which the *g*-factor is estimated from the data itself, and it can be readily applied to a broader range of datasets (but see Section 3.7 “Effect of *g*-factor estimation method” where the NORDIC.m flavor was explored).

### 2.3 Data analysis

NORDIC was applied to each run individually as the first preprocessing step. Aside from “gfactor_patch_overlap = 6” and “phase_filter_width = 10,” we used default parameters, including the default isotropic patch size: k × k × k, where $k={\left(\mathrm{numberOfTRs}\cdot 11\right)}^{1/3}\text{,}$ rounded to the nearest integer. For all versions, time series were then motion-corrected with AFNI (Cox, 1996) using identical transformations estimated from the “combined-withNoiseVolume-complex” version. This was done to avoid interactions between motion estimation and denoising performance (although motion estimates were largely identical across denoising versions, results not shown here). The motion-corrected time series were used to calculate BOLD-corrected VASO using LAYNII (Huber et al., 2021). Trial-wise percent signal changes (PSCs) were then calculated from the ROI-averaged time series as the mean of task blocks minus the mean of rest blocks divided by the latter (the first two volumes were removed from each block to account for intermediate signal values during transition periods). ROIs were manually delineated based on structural contrast in motor and visual brain patches that showed strong BOLD activation (Figure 2D). This approach is chosen to minimize the potential bias of excluding noise-dominated weak voxels in brain areas that are expected to be activated as a whole.

Relevant performance metrics (tSNR, *t*-values, and PSC) were extracted from a large ROI (>7,000 voxels) including visual and motor cortices (Figure 2D upper panel). Voxel-wise cortical depth estimates were obtained between manually segmented white matter and cerebrospinal fluid boundaries using LAYNII (data upsampled to 0.23 mm isotropic resolution). Laminar profiles reflecting response amplitude as a function of cortical depth were then extracted from a small ROI placed in V1 (Figure 2D lower panel). Note that the response amplitude here was quantified as the raw difference (referred to as delta) between the mean of task blocks and the mean of rest blocks (the first two volumes of each block were still discarded). That is, we refrained from normalizing by the baseline signal to avoid division by values close to 0, which could be a concern for the blood-nulled VASO signal, especially toward CSF. This was not an issue in the non-laminar analyses where the baseline signal was averaged across all ROI voxels before division.

### 2.4 Statistical analysis

Differences in the average tSNR, *t*-value, and PSC between versions were evaluated using paired two-sided *t-*tests. Correction for multiple comparisons was performed using the Holm–Bonferroni method (Holm, 1979). The significance level was set at α = 0.05.

## 3 Results and discussion

### 3.1 Degree of noise removal

**Figure 3:**
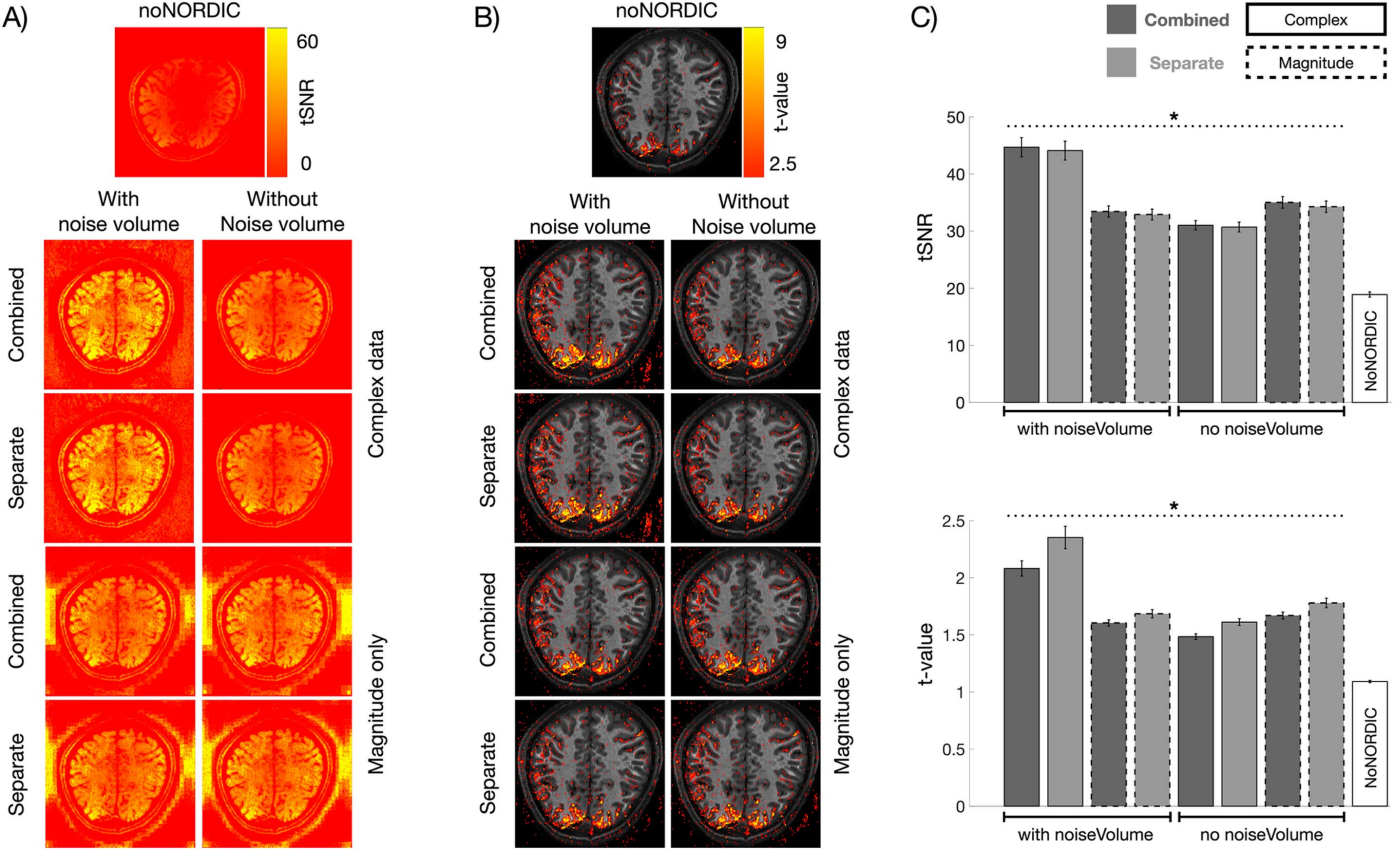
Evaluation of tSNR and *t*-value gains following denoising. **(A)** tSNR maps computed from nulled, motion-corrected, and detrended files. **(B)** *t*-values were computed across single-trial PSC estimates (*N* = 72). **(C)** Bar graphs representing ROI-averaged tSNR (upper) and *t*-values (lower) for all versions. For this plot, to illustrate the variability across runs, we computed absolute-valued *t*-scores across single-trial PSCs within each run (*N* = 12 per run). All versions had increased tSNR and *t*-values compared with noNORDIC (*p* < 0.05, denoted by dotted black horizontal lines and asterisks). The combined versions had higher tSNR on average than the separate versions (*p* < 0.05). This was reversed for *t*-values which were, higher on average for the separate versions than for the combined versions (*p* < 0.05). Error bars represent standard error across runs.

tSNR and *t*-value maps provide a measure of the degree of noise removal, with tSNR being a measure of temporal variance removed by NORDIC and *t*-values measuring whether the variance removed included relatively less task-locked signal of interest. As visible in Figure 3, these metrics were significantly increased in all denoised versions relative to noNORDIC. The complex-valued versions with the appended noise volume stood out with the largest gains, whereas the remaining versions were close (Figure 3). Even for these latter versions, NORDIC increased tSNR and the average single-voxel *t*-value by approximately 50%, which can be translated to, e.g., shorter scan durations, higher resolutions, or lower CNR. Interestingly, the “combined” versions yielded significantly higher tSNR than the “separate” versions, suggesting more noise removal. However, this came at the cost of increased signal loss, as evident from the reversal of this pattern in the *t*-values (i.e., the combined versions had significantly smaller *t*-values on average compared with the separate versions). This point is further underscored in the PSC evaluation detailed in “3.2 Response amplitude intactness after denoising.” Thus, the “separate-withNoiseVolume-complex” version seemed to provide the most efficient denoising based on tSNR and *t*-values alone.

The large tSNR values outside the brain for magnitude-only versions could be an effect of denoising in areas with low SNR where the noise distribution is non-Gaussian for magnitude data. In this Rician noise regime, the thermal noise distribution is a function of signal intensity (Gudbjartsson and Patz, 1995), and the spectral distribution of eigenvalues in the PCA decomposition is less distinct, whereby signal fluctuations are more likely removed. This would mean that the NORDIC assumption of Gaussian-distributed noise is not maintained, resulting in too high noise thresholds. In any case, it does not seem to be an issue inside the brain.

### 3.2 Response amplitude intactness after denoising

**Figure 4:**
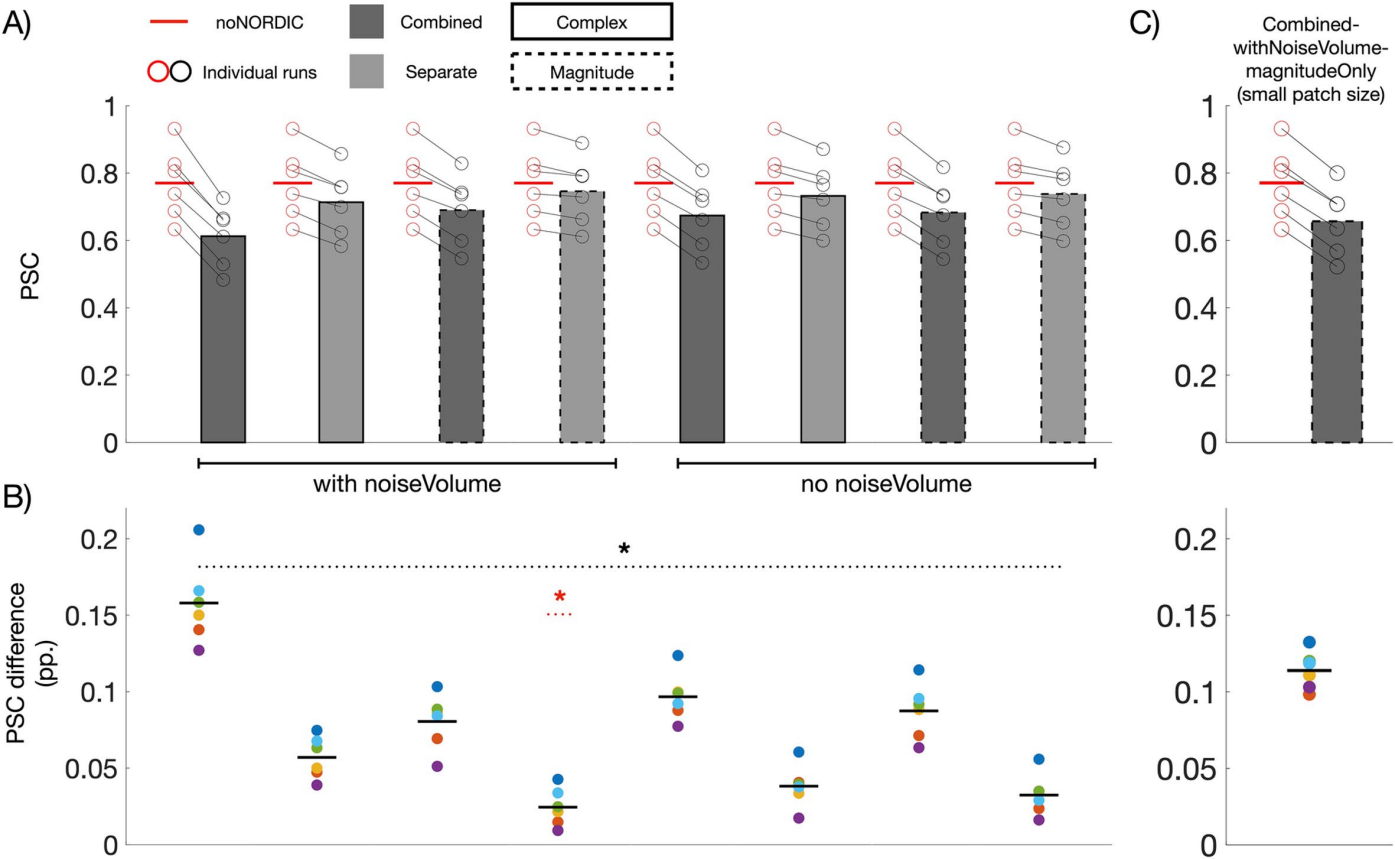
Effect of NORDIC on response amplitude. **(A)** Voxel- and run-averaged PSCs with and without denoising (represented with bars and horizontal red lines, respectively). Circles denote the PSC of individual runs. All versions had a reduced PSC compared with noNORDIC (*p* < 0.05, denoted by a dotted black horizontal line and asterisk). This effect was larger on average for the combined versus the separate versions (*p* < 0.05). **(B)** Colored circles depict the run-wise differences in PSC between noNORDIC and NORDIC (red circles minus black circles in **A**). Black horizontal lines represent the mean across runs. The “separate, with noise-vol, magnitude-only” version had significantly smaller PSC reduction than each of the other versions (*p* < 0.05, denoted by a dotted red horizontal line and asterisk). **(C)** Same as “combined-withNoiseVolume-magnitudeOnly” in **A** but using a smaller patch size.

If NORDIC only removes thermal noise, the average PSC across ROI voxels should be unaltered (the ROI has over 7,000 voxels, and thermal noise should thus be practically eliminated after averaging, albeit a small bias will be present if the mean of the noise is non-zero). Figure 4A shows bar plots of PSCs computed from average time series across voxels in the ROI. All versions had a significantly reduced PSC compared with noNORDIC. In line with the conclusion from Figure 3C, this effect was on average significantly larger for combined versions compared with the separate versions (Figure 4B), suggesting that NORDIC should preferably be performed on nulled and not-nulled time series separately. For the separate versions, the effect was quite small, particularly for “separate-withNoiseVolume-magnitudeOnly,” which had a significantly lower PSC reduction than each of the other versions (Figure 4B). Since the patch size depends on the number of timepoints, we considered the possibility of the patch perhaps being over-sized for combined versions. However, the bias prevailed even when the patch size was set to something smaller (28×28×1, *N* = 784) than for the separate versions (*N* = 1728) (Figure 4C).

**Figure 5:**
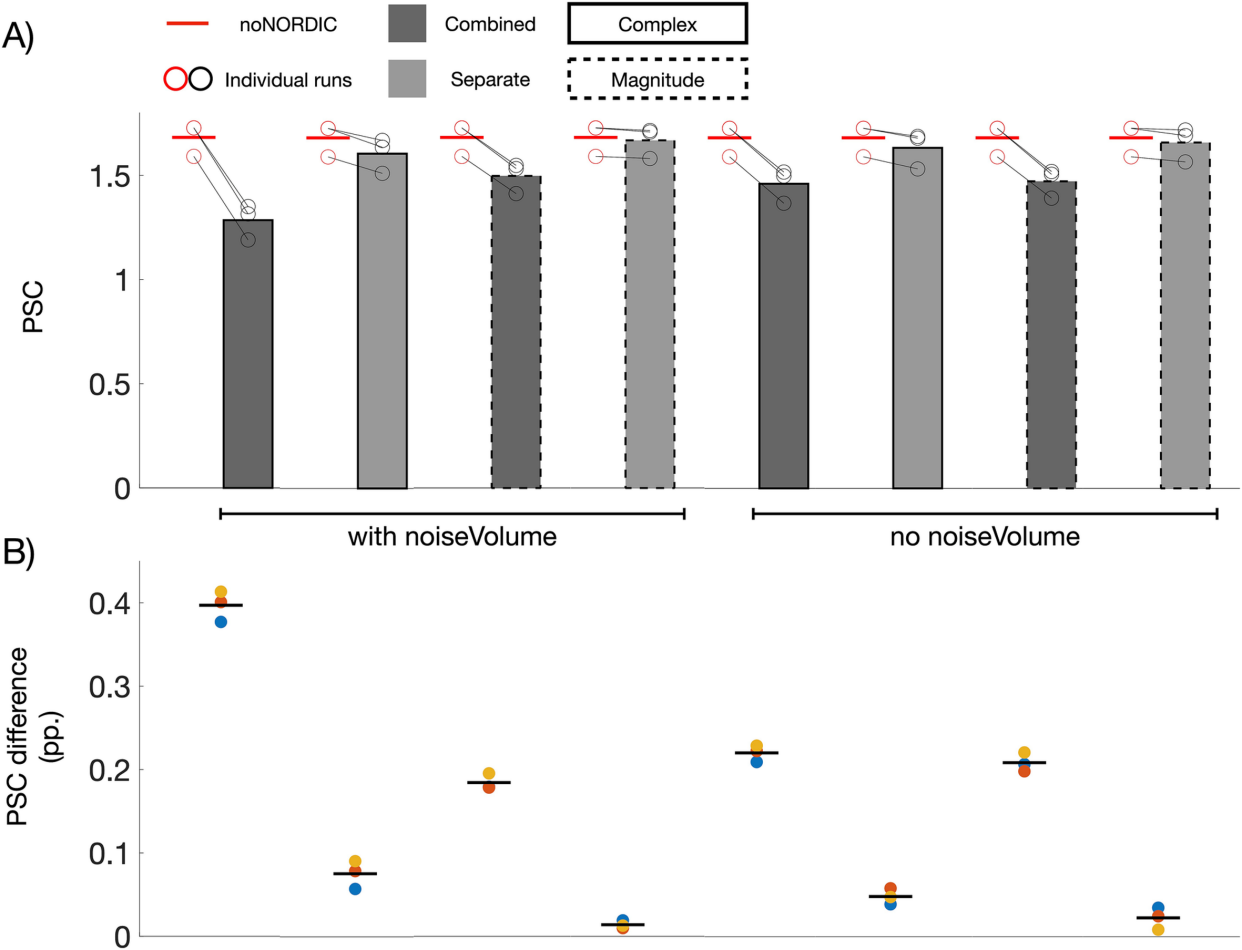
Replication of Figure 4 using voxels (*N* = 1,463) within the ROI with *t*-values >2 based on noNORDIC trials from the first three runs. Red (noNORDIC) and black (NORDIC) circles in **A** represent PSCs of the last three runs. The corresponding run-wise PSC differences between noNORDIC and NORDIC are represented by colored circles in **B**.

With the above approach to assess bias, signal removal could be masked if the PSC is biased toward 0 for both positively and negatively responding voxels. This motivated a replication of Figure 4 using a split-half approach across runs. Here, we only included within-ROI voxels showing a somewhat clear positive response (*t*-values >2 based on the first three runs). In line with observations in Figure 4, all versions showed a consistently reduced PSC (across the last three runs) compared with noNORDIC, with the largest bias in the combined versions (Figure 5). For the separate versions, the PSC bias appeared similar to or even reduced compared to Figure 4, suggesting that, also when accounting for this masking effect, NORDIC only minimally biased response amplitudes, particularly in the “separate-withNoiseVolume-magnitudeOnly” version.

### 3.3 Intactness of temporal structure

**Figure 6:**
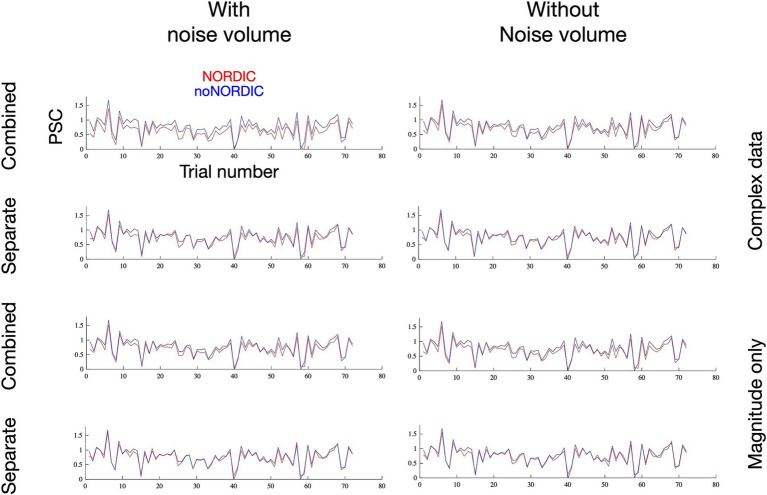
Single-trial percent signal change as a function of trial number following thermal noise suppression through extensive voxel averaging. The remaining trial-by-trial variance, assumed to reflect “true” signal variability and physiological noise sources, appears to be contained after NORDIC as desired.

In addition to testing for changes in response amplitude, we further evaluated whether NORDIC altered the temporal structure of single-trial responses (Figure 6) by plotting the voxel-averaged PSC as a function of trial number. The versions with the largest bias in PSC based on Figures 4, 5 naturally had a reduced mean. However, the temporal pattern of trial variability seems largely unaltered, as supported by the correlation coefficient between NORDIC and noNORDIC trial time series being >0.95 for all versions. If NORDIC altered the variability across trials (reduced variability is expected at the single-voxel level but not after averaging across >7,000 voxels), it would bias *t*-values (on top of the bias induced by response amplitude alterations), but that was not the case. The non-thermal “true” trial variance in response amplitude, originating, for example, from differences in attention, movement kinematics, and compliance, thus appears to be well-preserved.

### 3.4 Intactness of spatial structure

**Figure 7:**
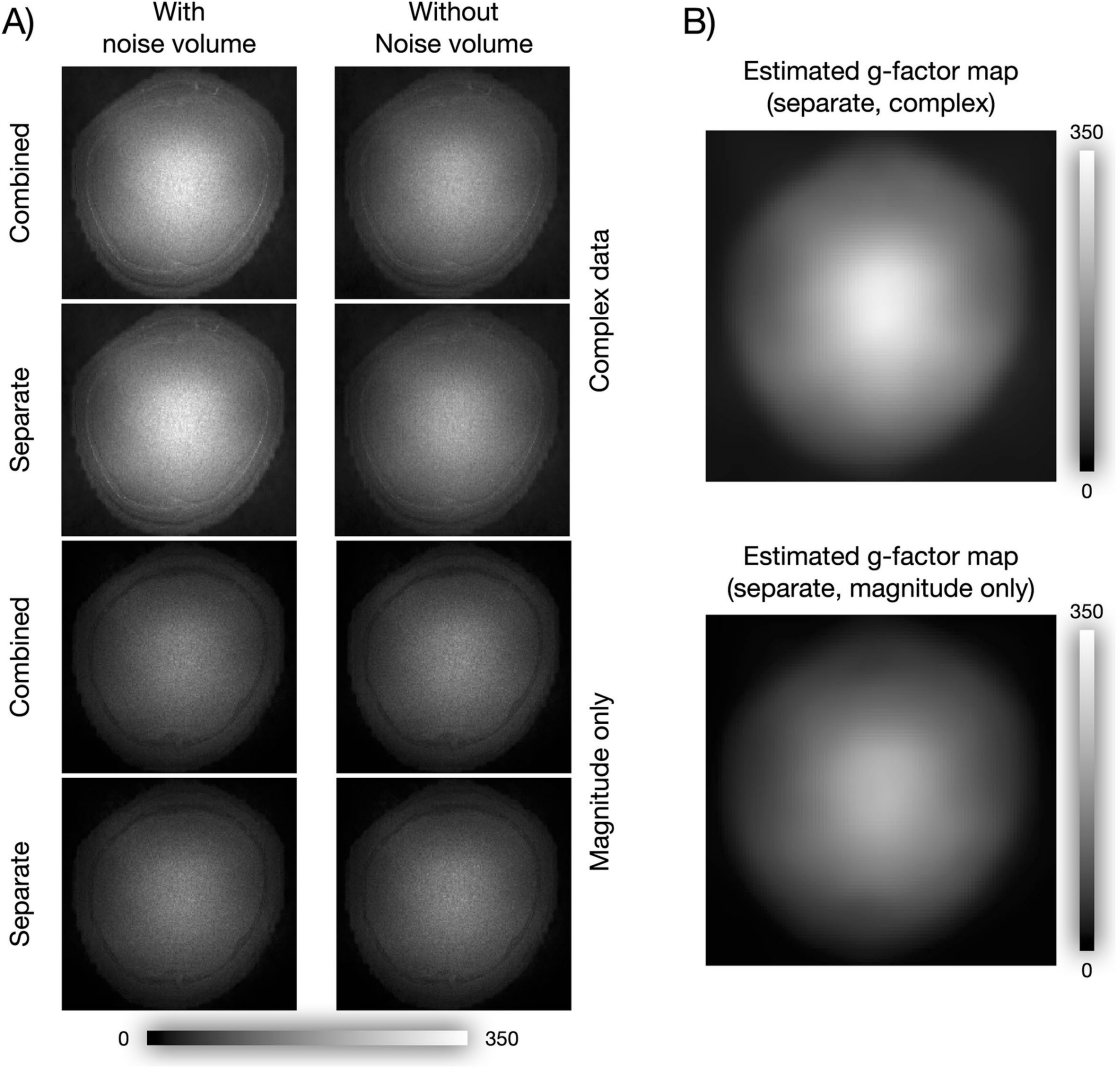
Did NORDIC spatially blur the data? **(A)** Mean images of NORDIC minus noNORDIC time series. For complex images, the difference was computed as the absolute value of the complex difference, and for magnitude-only images, it was computed as the absolute value of the magnitude difference. **(B)** *g*-factor maps estimated by two different NORDIC versions, shown here to illustrate that the mean of the difference time series in **A** appears as non-structured random noise scaled by the *g*-factor.

To test whether NORDIC compromised spatial resolution, we computed an image of the mean nulled difference time series (first run), i.e., NORDIC minus noNORDIC, reflecting what is removed by NORDIC (Figure 7A). It should be noted that while all versions seem to have some structure in the periphery, the difference images within the brain look like unstructured random noise scaled by the *g*-factor as expected if NORDIC did not introduce blurring (Figure 7B).

### 3.5 Laminar profiles

**Figure 8:**
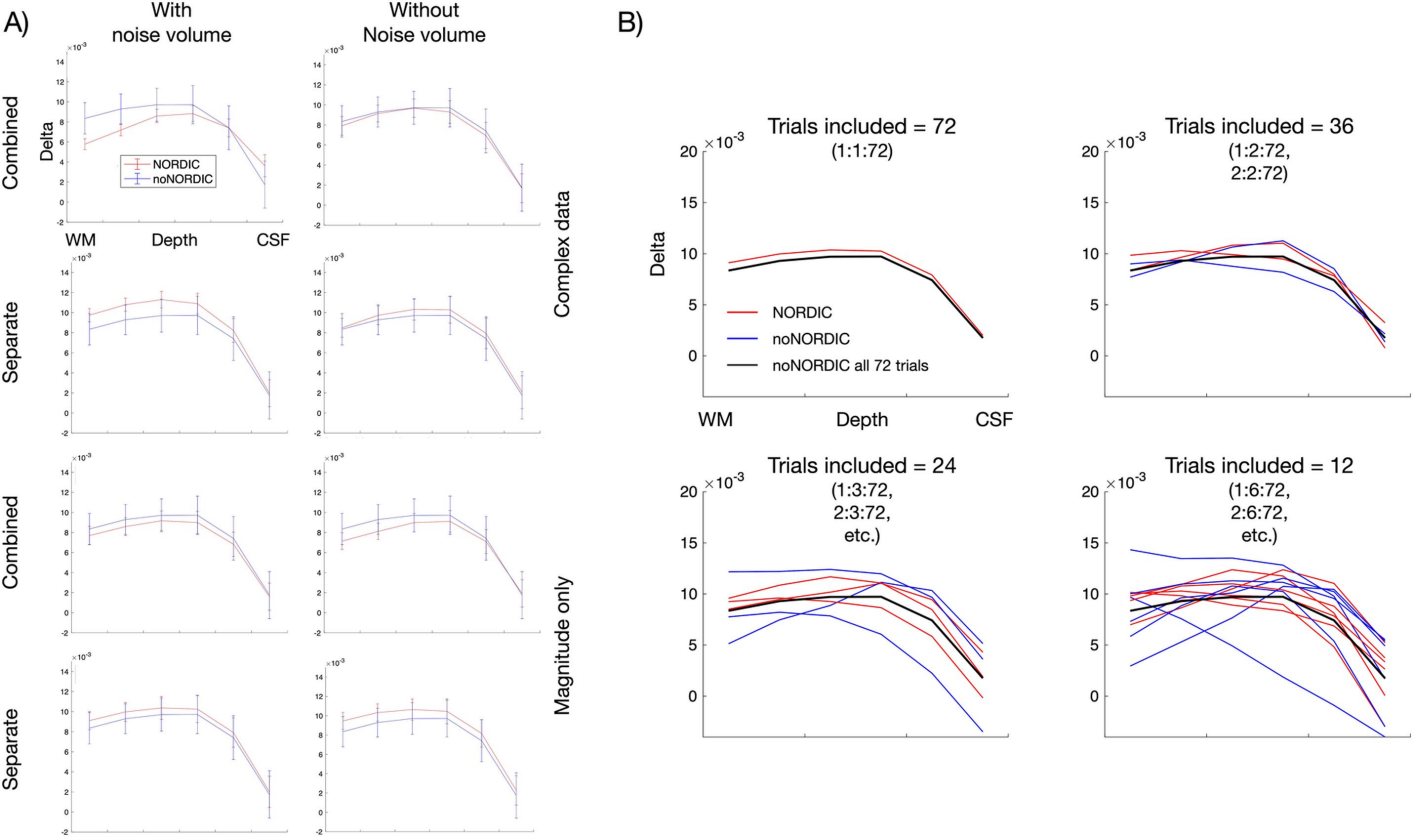
Layer-dependent signal preservation with NORDIC. **(A)** Laminar profiles for each version obtained from the layered ROI shown in Figure 2D. **(B)** Laminar profiles with different subsampling schemes for the “separate, with noise-vol, magnitude-only” version. For plot 1.1, trials 1:1:72 were averaged; for plot 1.2, one of the profiles was averaged across trials 1:2:72 (2:2:72 for the other), etc. Note that these layer-fMRI profiles represent a relatively small cortical patch (Figure 2D) that is manually selected to be (i) a sulcus inside the brain without noise transitions between tissue and skull and (ii) not affected by large pial vessels that might have residual intravascular BOLD fluctuations at 3T.

Finally, we evaluated the spatiotemporal signal intactness after NORDIC based on laminar analysis. Figure 8A shows laminar profiles of each version—if denoising only removed thermal noise, NORDIC profiles should converge with the noNORDIC profile (after averaging 72 trials) in terms of response amplitude and shape, but with smaller error bars. This seems to be fulfilled for all versions (except the “combined, with noise-vol, complex” version), suggesting that robust estimation of laminar profiles could be obtained with fewer trials when using NORDIC. This is illustrated in Figure 8B (for the “separate-withNoiseVolume-magnitudeOnly” version), showing profiles with different degrees of trial subsampling. Subsampled noNORDIC profiles start breaking down already at 24 trial averages, whereas NORDIC profiles seem quite stable even at 12 averages. Furthermore, the observation of preserved profile shapes supports the conclusion in Section 3.4 that denoising did not result in noteworthy spatial blurring of the data.

In summary, the version that seemed to best preserve the signal was the “separate-withNoiseVolume-magnitudeOnly” (wrapper for running this version can be downloaded here: https://github.com/LasseKnudsen1/NORDIC-VASO), closely followed by the “separate-withoutNoiseVolume” versions, none of which were the ones providing the largest noise removal. This highlights that denoising performance should not be evaluated solely from the amount of noise removal; continued careful evaluation of signal intactness is critical. Importantly, more noise removal is often followed by more signal removal, thus representing a trade-off between the two (Kay (2022) for an excellent illustration), which we will look into in the following section.

### 3.6 Manipulating the trade-off between noise removal and signal preservation

**Figure 9:**
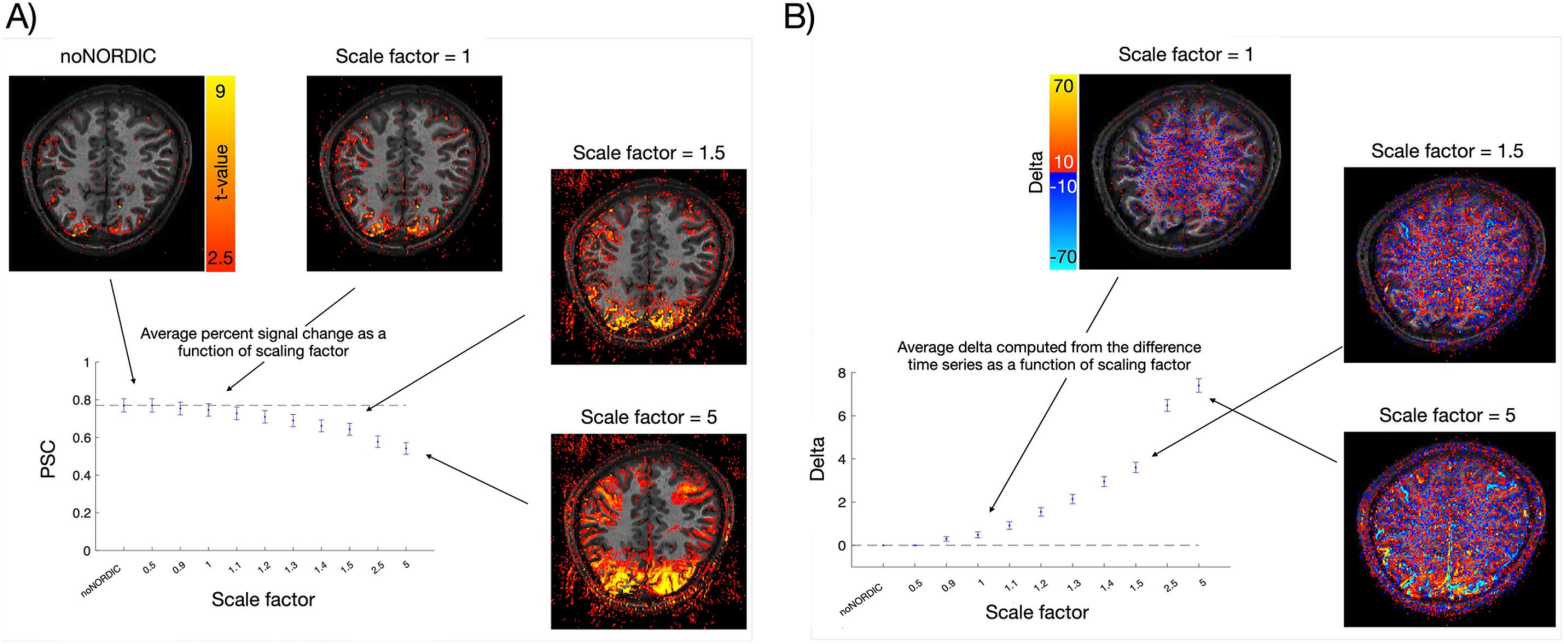
Illustrating the trade-off between noise removal and signal preservation. **(A)** Average VASO PSC across ROI voxels and trials for different scaling factors with corresponding *t*-value maps. The dashed line represents the mean PSC of noNORDIC. Error bars reflect standard error across trials. **(B)** Across-trial-averaged delta (mean of stimulation blocks minus mean of rest blocks) computed from the noNORDIC minus NORDIC difference time series with corresponding delta activation maps. Note that since difference time series cannot readily be BOLD-corrected, we used not-nulled (BOLD) time series for this plot. The dashed line represents a delta of 0 as expected if no signal is removed. Error bars reflect standard error across trials.

Based on the analyses above, we prefer the “separate-withNoiseVolume-magnitudeOnly” version as it seemed to be the best performing in terms of removing noise while keeping the signal intact. Accordingly, we chose to move on with this version for the next analysis. Here, we denoised the data with different values of NORDIC’s “factor_error” parameter. The default value is 1, and it simply scales the originally determined singular value threshold. That is, if “factor_error = 1.2,” the threshold is increased by 20% and more components will be removed, resulting in more aggressive denoising. The risk, of course, is a speculated increased likelihood of concurrently removing the signal of interest or imposing temporal signals. Figure 9A illustrates this by showing the ROI-averaged PSC as a function of the scaling parameter. Increasingly more signals might be speculated to be removed as we increase the scaling factor, but the overcompensating noise removal (possibly also including physiological components at higher scale factors) leads to robust sensorimotor activation in the activation maps (*t*-values), which was not clear at the default scaling of 1 (some of which may also reflect spatial blurring at higher scale factors). To further emphasize the point of adverse signal removal, we computed delta activation maps from the difference time series (noNORDIC minus NORDIC), i.e., the part removed by NORDIC. Figure 9B shows how the difference time series (computed from not-nulled data as explained in the caption) contains increasingly more signals when the scaling factor grows.

With this parameter, we can balance the trade-off between noise and signal removal as desired. For example, it may be useful to push the scaling factor for functional localizers where we do not care so much if the response amplitude is accurate; we just want to find the right region—in this way, the acquisition time spent on localizer runs could be dramatically shortened, and one has more time to do the actual experiment. Another example would be the acquisition of a distortion-matched T1-weighted anatomical reference computed from a VASO time series, where alterations of the signals do not really matter as long as the anatomical structure is intact. The right choice of picking an optimal threshold value in these speculated application cases might require extra care, validation, and training, comparable to the challenges in ICA denoising of fMRI data.

The fact that the PSC is already slightly but significantly reduced at a scaling factor of 0.9 compared with noNORDIC (paired *t*-test across trials, *t* (71) = −2.94, *p* = 0.004) may reflect an important issue for all low-rank denoising methods: in the presence of a noise level that is comparable to the signal magnitude, the estimated principal components will always deviate from the noise-free scenario. That is, estimated components will be “corrupted” to some extent such that thermal noise and signal end up being spread out across all components (Kay, 2022; Olesen et al., 2023). In noisy experimental environments, the effect is always there, and it only becomes a matter of its extent. In many cases, it may be negligible, and a lot of noise removal at the cost of a very small bias will be well worth it, especially in cases where no activation is detectable without denoising.

With respect to the 3T data investigated here, we do find it promising that even for the present dataset where CNR is fairly low (3T VASO at submillimeter resolution), the signal reduction appears to be quite small at the default scaling factor and when the implementation of NORDIC is tailored for VASO. This was also the case in two additional VASO datasets with different field strength (7T), sequence parameters, tasks, number of volumes, participants, etc. The results from one of these datasets are shown in the section below, and the other is from the findings of the study by Huber et al. (2017) (results not shown here, but data can be downloaded at: 10.6084/m9.figshare.26819422).

### 3.7 Effect of *g*-factor estimation method

The MATLAB implementation of NORDIC can either use the *g*-factor map provided by the scanner (NORDIC.m), which was computed here by the vendor’s reconstruction in IcePat, or the *g*-factor can be estimated from the input time series (NIFTI_NORDIC.m). The former should be closer to the ground truth and may therefore be preferable. To test this for VASO, we ran a similar analysis as presented in Sections 3.1–3.5, but this time on a 7T dataset (0.8 mm isotropic, six runs of bilateral finger tapping (two of which were discarded due to motion), 12 min each), where scanner-provided *g*-factor estimates were available.

To test whether it is worthwhile to obtain a *g*-factor map for each run (which can be impractical and time-consuming) or whether it is sufficient to reconstruct a single map at the beginning of the session, we ran NORDIC either with the same *g*-factor map for all runs (first run) or with run-specific maps. The current version of NORDIC.m can only be applied to complex data and with appended noise volumes. Thus, to most directly evaluate the effect of actual vs. estimated *g*-factors, we also ran NIFTI_NORDIC.m with appended noise volumes and on complex data. To test whether these versions were preferable compared with the version recommended based on Sections 3.1–3.5, we additionally ran NIFTI_NORDIC.m with appended noise volumes but on magnitude-only data. All versions were denoised on nulled/not-nulled time series separately as deemed optimal based on Sections 3.1–3.5.

**Figure 10:**
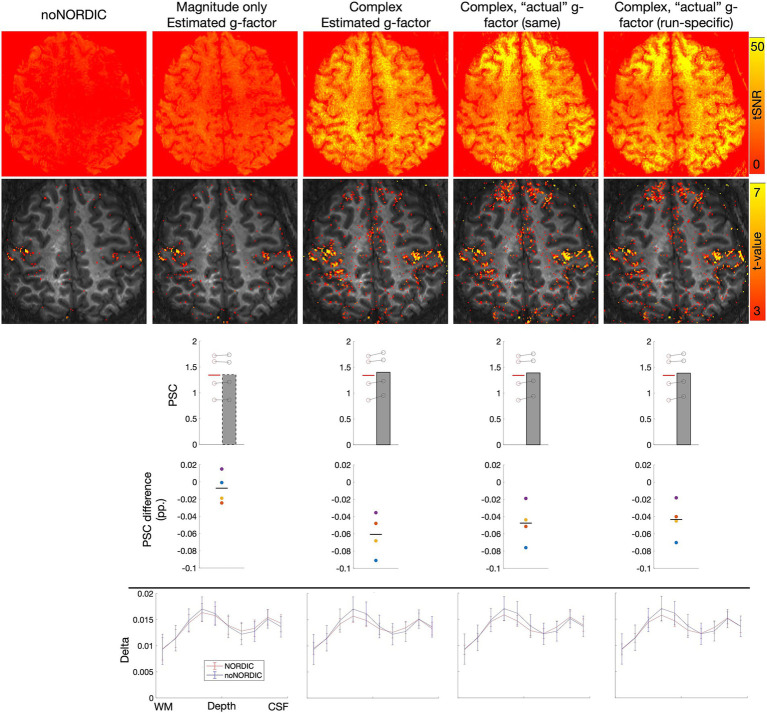
Effects of the *g*-factor estimate on denoising performance. Same types of plots as in Sections 3.1–3.5. Columns 2–3 depict results from the NIFTI_NORDIC.m versions (magnitude-only or complex-valued, respectively). Columns 4–5 depict results from the NORDIC.m versions (using the same *g*-factor map from the beginning of the session for all runs or run-specific maps, respectively). For all versions, denoising was performed with appended noise volumes and on separate nulled and not-nulled time series.

**Figure 11:**
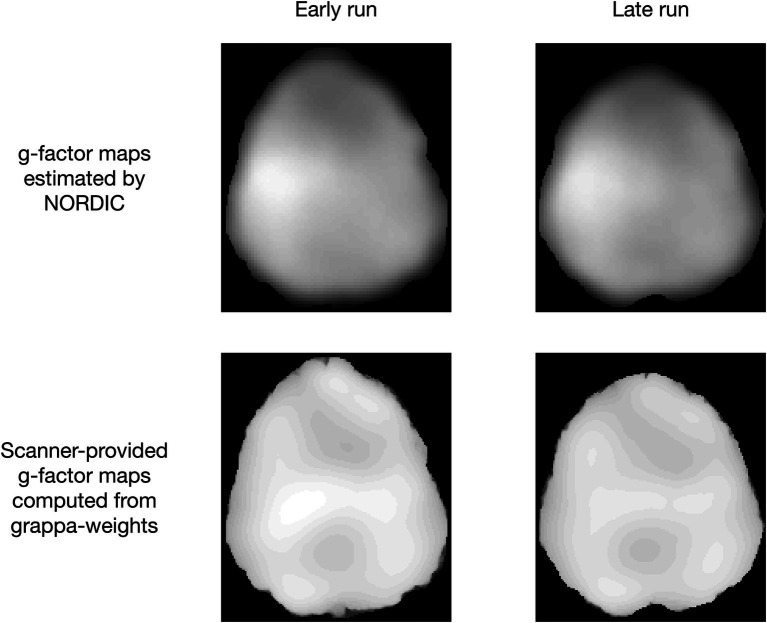
Brain-masked *g*-factor maps either estimated by NORDIC using MP-PCA (Veraart et al., 2016a, 2016b) or estimated using the vendor’s reconstruction in IcePat. Note that the motion-induced difference across runs only minimally affected the denoising based on Figure 10.

All results (Figure 10) were extracted from a large ROI covering the hand knob of M1 in both hemispheres (ROI for laminar profiles only covered the hand knob in the left hemisphere). First, tSNR and *t*-value maps, PSC evaluations, and laminar profiles appear highly similar between using the same or run-specific *g*-factor maps, suggesting that one does not necessarily have to bother with computing *g*-factor maps for every run. This was the case despite large across-run motion, which resulted in noticeable *g*-factor differences across runs (Figure 11). Second, the same can be said when comparing NORDIC.m versions with the complex-valued NIFTI_NORDIC.m version, suggesting that denoising is not wildly sensitive to (small) inaccuracies in *g*-factor estimates (although some local differences can be seen, for example, in the tSNR maps). Third, all these versions had signs of signal alteration, as visible in the bar plots. This problem was largely gone for the NIFTI_NORDIC.m “separate-withNoiseVolume-magnitude-only” version (the “winner” from Sections 3.1–3.5), but that version also had less noise removal (~50% tSNR increase compared to >100% increase for the complex versions).

### 3.8 Key takeaways and potential future extensions

VASO-specific findings were:

- While denoising nulled/not-nulled time series combined provides more time points and principal components, with the potential to better separate signal from thermal noise, the strong contrast difference may ultimately introduce counter indications that make the separation harder for PCA.
- As for conventional BOLD, an appended noise volume in the recommended implementation improved the performance of NORDIC.
- We found the unique phase behavior in inversion–recovery VASO with positive and negative phases (longitudinal magnetization directions) may call for a conservative application of NORDIC with magnitude-only data. This is different from the application of BOLD-only data, where complex-valued NORDIC is advised (Vizioli et al., 2021).

Potential points in future studies to evaluate, optimize, and extend NORDIC:

- If NORDIC removes part of the signal, then averaging across runs still has some advantages compared to noNORDIC. This would also mean that combining the two approaches does not benefit you twice in some cases.
- In single-participant analysis, the reduction in effect size (*β*, PSC) can come with a much larger reduction in variability—this is why in the beginning, we see that despite the drop in PSC, the “combined” versions gave the largest benefit in *t*-values. However, in multi-participant analyses, this issue might be more tricky. The reduction in effect size will not always be paralleled by a stronger reduction in variability as NORDIC does not necessarily strongly affect the variability across subjects. Thus, the reduction in percent signals can be problematic, as it can affect differences between task conditions. Seen this way, NORDIC has all the same bias-variance trade-offs present in many other processing and denoising tools. It reduces variance in the individual dataset at the cost of some bias (toward 0). In general, reducing noise is advantageous, but there is a point where too much bias becomes an issue (think of typical machine learning examples).
- We believe that it could make sense to focus future extensions of NORDIC onto the interaction of the design with the NORDIC efficiency. It might become advantageous to solely remove principal components that have zero correlation with the task design. However, such an approach might come along with further implications, for example, what happens to transit responses in block design studies after NORDIC?
- Overall, the more complicated endeavor is to make sure that the removed signal is not biased in some way between conditions (i.e., removing more of one condition compared to another). Based on the results shown here, there might be potential mechanisms in which this could happen at some point. This would mean that NORDIC would not only remove a part of the main effect but also affect the differential effects more than just making them smaller.

## 4 Conclusion

Based on the present evaluation, we suggest that, for VASO, NORDIC is executed on nulled and not-nulled time series separately, that a noise-only volume is included, and that only the magnitude data is used. This version seemed to best preserve the underlying signal and did come with a large reduction in thermal noise, thus alleviating CNR limitations in submillimeter VASO acquisitions, which may be particularly valuable at lower field strengths. Future extensions of these evaluations across more participants and more experimental setups will help to increase the generalizability of these findings. The study conducted here paved the way for sensitive, vein-free submillimeter fMRI across clinical field strengths and 7T.

## Data Availability

The 3T data are available at: https://layerfmri.page.link/ME_VASO3T. The 7T data are available at: 10.6084/m9.figshare.26819422. The analysis code and a wrapper for running NORDIC on VASO data are made available at: https://github.com/LasseKnudsen1/NORDIC-VASO.

## Footnotes

^1^https://github.com/SteenMoeller/NORDIC_Raw, v1.1, commit 74999d6.
